# Mothers' satisfaction with referral hospital delivery service in Amhara Region, Ethiopia

**DOI:** 10.1186/1471-2393-11-78

**Published:** 2011-10-24

**Authors:** Azmeraw Tayelgn, Desalegn T Zegeye, Yigzaw Kebede

**Affiliations:** 1 Department of Public Health, Bahir Dar University, P.O.Box 79, Bahir Dar, Ethiopia; 2 Department of Epidemiology and Biostatistics, University of Gondar, P.O.Box 196, Gondar, Ethiopia

## Abstract

**Background:**

A woman's satisfaction with the delivery service may have immediate and long-term effects on her health and subsequent utilization of the services. Providing satisfying delivery care increases service utilization. The objective of this study is to assess the satisfaction of mothers with referral hospitals' delivery service and identify some possible factors affecting satisfaction in Amhara region of Ethiopia.

**Methods:**

A hospital-based cross-sectional survey that involved an exit interview was conducted from September to November 2009 in three referral hospitals in Ethiopia. A total of 417 delivering mothers were enrolled in the study. Client satisfaction was measured using a survey instrument adopted from the Donabedian quality assessment framework. We collect data systematically from every other postnatal woman who delivered in the referral hospitals. Multivariate and binary logistic regression was applied to identify the relative effect of each explanatory variable on the outcome (satisfaction).

**Results:**

The proportion of mothers who were satisfied with delivery care in this study was 61.9%. Women's satisfaction with delivery care was associated with wanted status of the pregnancy, immediate maternal condition after delivery, waiting time to see the health worker, availability of waiting area, care providers' measure taken to assure privacy during examinations, and amount of cost paid for service.

**Conclusions:**

The overall satisfaction of hospital delivery services in this study is found to be suboptimal. The study strongly suggests that more could be done to assure that services provided are more patient centered.

## Background

One of the Millennium Development Goals (MDG5) is to reduce the maternal mortality ratio by 3/4 between 1990 and 2015. Pregnancy and childbirth claim the lives of an estimated half a million of women globally each year [1]. More than half of these deaths occur in Africa [1]. Ethiopia is one of the countries that have highest maternal mortality rates (MMR) in the world which is estimated to be 673/100,000 live births [2]. Part of this mortality is attributed to poor delivery care [3].

The Ethiopian government and international organizations are working for making hospital delivery services accessible and usable for all pregnant women but still the proportion of births attended by a skilled birth attendant is about 18.4% [4] in 2009 which was much lower than the average level in developing countries in general (59%), Sub-Saharan regions (44%), and very far from MDG target of 90% coverage [1,3]. The reasons forwarded by researchers for the higher maternal mortality, and lower coverage of skilled delivery in Ethiopia include mothers' unhappy health institutional delivery experience [3,5].

In Ethiopia, dire health institutional delivery experience limits women's ability to seek care for subsequent pregnancies [3,5]. A midterm assessment of the third Ethiopian Health Sector Development Program (HSDP III) also identified poor quality skilled birth attendance as one of the critical bottlenecks for improving the lower utilization of hospital delivery [5]. Many studies have been conducted on quality of maternity care with hospital and health centers in Ethiopia [6,7]. These studies suggest that the quality of maternity care in Ethiopia is poor. This includes not only the quality of clinical care but also gender sensitivity, preservation of dignity and cultural sensitivity [5,7]. These factors together with the community level factors explain the extremely low utilization of health services for delivery [3,6,7].

Satisfaction is a meaningful output indicator of quality health care [8-10]. Various studies have reported that satisfied service users are more likely to utilize health services, comply with services and follow ups, and continue with the health care [11-14]. Satisfaction with childbirth experience is important to the woman, infant's health and well-being, and mother-infant relationship. Studies reported that a mother's positive perception of birth experience has been linked to positive feelings toward her infant and adaptation to the mothering role [15-17].

A community based study conducted in Kenya among women who delivered in health facilities showed that over half (56%) of women are satisfied with delivery care [11].

Despite having many studies done elsewhere, there is paucity of data concerning mother's satisfaction with childbirth services in referral hospitals in Ethiopia. Hence, this study was conducted to show Ethiopian case at referral level in Amhara region of Ethiopia.

## Methods

### Study design

A hospital-based cross-sectional survey was conducted from September 2009 to November 2009.

### Study area

This study was conducted in three public referral hospitals of Amhara region, northwest Ethiopia. These three referral hospitals were University of Gondar Teaching and Referral Hospital (UOGTRH), Felege Hiwot referral hospital (FRH), and Dessie referral hospital (DRH). Each referral hospital's catchment population is estimated to be 5-7 million people.

### Study population

Selected mothers who visited the three referral hospitals for delivery service during the data collection period were the study population.

### Sample size

The sample size for this study was determined using single population proportion formula considering the assumptions: Proportion of delivering mothers satisfied with hospital delivery care service as 56% (**p **= 0.56) [11]. Level of significance to be 5% (α = 0.05), **Z α**/***2 ***= 1.96 and margin of error to be 5% (**d **= 0.05). Adding non responses rate of 10%, a total sample size of 417 delivering mothers were included.

### Sampling Procedure

The total sample size was allocated proportionally to each of the hospitals by reviewing the number of deliveries attended in the 1^st ^and 2^nd ^quarter of 2009. Data were collected from every other postnatal woman who received delivery care in the three hospitals.

### Data collection instrument

The data collection instrument was closed-ended questionnaire. Delivery service satisfaction related questions were adopted from the Donabedian quality assessment framework [8] and presented using a 5- scale likert scale (1-very dissatisfied, 2-dissatisfied, 3-neutral, 4-satisfied, and 5-very satisfied).

The first draft of the English questionnaire was translated to Amharic language by independent translators then back to English language to check for consistency. The questionnaire was pre tested on 40 postnatal mothers at the University of Gondar Teaching and Referral Hospital one month prior to the actual data collection.

### Data collection

Six female nurses who were fluent in Amharic and who were not working in the study sites were recruited for data collection. Three days (two days theoretical and one day practical) training was given before the actual data collection.

### Data analysis

The completed questionnaires were checked for completeness and consistency by the investigators. Data was entered, cleaned and analyzed using SPSS version 16 (SPSS Inc., Chicago). During analysis, the responses of 'very satisfied' and 'satisfied' were classified as satisfied and responses of 'very dissatisfied', 'dissatisfied' and 'neutral' as unsatisfied. Neutral responses were classified as dissatisfied considering that they may represent a fearful way of expressing dissatisfaction. This is likely because the interview was undertaken within the hospitals and mothers may have been reluctant to express their dissatisfaction feeling of the services they received.

For the overall satisfaction level, those who were satisfied in greater or equal to 75% of the items were categorized under "satisfied" and those who were satisfied in less than 75% of the items were categorized as "un satisfied". Descriptive statistics was computed for the study variables. Frequency distribution tables were used to describe most of the findings and graphs were also plotted for some. To determine factors associated with mother's satisfaction bivariate binary logistic regression and multivariate stepwise logistic regression were applied. Adjusted odds ratio was used to determine the strength of association between selected variables.

### Ethical considerations

The study proposal was approved by Institutional Review Board of the College of Medicine and Health Sciences of the University of Gondar. Informed oral consent was obtained from each study participant. Confidentiality was assured by making the questionnaire anonymous.

## Results

### Socio-demographic characteristics

A total of 417 delivering mothers from three referral hospitals participated in the exit survey with 146 (35%) of the women obtained from the UOGTRH, 150(36%) from FRH and the rest 121(29%) from DRH.

The mean ± SD age of the mothers was 25.9 ± 5.2 years. One hundred and twenty eight (30.7%) were with no formal education. The majority (89.2%) were married. One hundred thirty five (32.3%) were house wives while 86(20.7%) were government employees. Two hundred ninety six (71%) mothers came from urban areas. Three hundred and forty (81.5%) were Amhara by ethnicity. About three fourth (302) of the delivering mothers were Orthodox Christians by religion (Table 1). The median household income of the delivering mothers was 765 ETB (47.8USD). Three hundred ninety one mothers (98.3%) paid for the service received. The average payment by mothers was 157 ETB.

**Table 1:** Socio Demographic Characteristics of delivering mothers in referral hospitals of Amhara Region, Ethiopia, September - November 2009 (n = 417)

	Total(n = 417)
Characteristics	N (%)
**Age (in years)**	
< 20 years	37(8.9)
20-34 years	350(83.9)
35-49 years	30(7.2)
**Marital status**	
Married	372(89.2)
Single	39(9.4)
Divorced	6(1.4)
**Ethnicity**	
Amhara	340(81.5)
Tigray	36(8.7)
Oromo	9(4.5)
Agew	6(1.4)
Others	16(3.9)
**Religion**	
Orthodox	302(72.5)
Muslim	107(25.6)
Protestant	8(1.9)
**Educational Status**	
No formal education	128(30.7)
Grade1-6	69(16.5)
Grade7-12	158(37.9)
Above-12	62(14.9)
**Occupation**	
House wife	135(32.3)
Government employee	86(20.7)
Farmer	78(18.7)
Merchant	51(12.2)
Student	42(10.1)
Others	25(6.0)
**Residence**	
Urban	296(71.0)
Rural	121(29.0)
**Hospital delivery took place**	
UOGTRH	146(35)
FRH	150(36)
DRH	121(29)

### Obstetrics characteristics of delivering mothers

For 47.5% of women, this was the first delivery, 42.2% had had 2-5 deliveries; and 10.3% of women had had 5 or more deliveries. Nearly a quarter of women had had an unwanted birth. About 64.5% of the mothers' do not have previous health facility delivery experience. The majority (84.2%) had one or more ANC visits and 15.8% did not attend ANC. Over a fifth of women (21%) had a self-reported complication in pregnancy. Normal vaginal delivery was the commonest mode of delivery (53.7%) followed by assisted delivery (24.5%) and caesarean section (21.8%) (Table 2).

**Table 2:** Obstetrics characteristics of respondents in referral hospitals of Amhara Region, Ethiopia, September-November 2009(n = 417)

Obstetric characteristics	Total (n=417) N(%)
**Parity (number including the new baby)**	
One	198(47.5)
Two to five	176(42.2)
More than five	43(10.3)
**Reason for visit**	
Planned delivery	342(82.0)
Referral delivery	75(18)
**Wanted status of pregnancy**	
Wanted	308(73.9)
Unwanted	109(26.1)
**Mode of delivery**	
Spontaneous vaginal delivery(SVD)	224(53.7)
Assisted delivery	102(24.5)
Caesarean section(C/S)	91(21.8)
**Immediate maternal condition after delivery**	
Normal	328(78.7)
With complications	89(21.3)
**Fetal outcome**	
Live birth	374(89.7)
stillbirth	43(10.3)
**ANC follow up**	
Yes	351(84.2)
No	66(15.8)

### Mothers' satisfaction on delivery care

The proportion of mothers who were satisfied with delivery care in this study was 61.9%. Dissatisfaction was highest (74.7%) among women delivering at UOGTRH.

**Figure 1:**
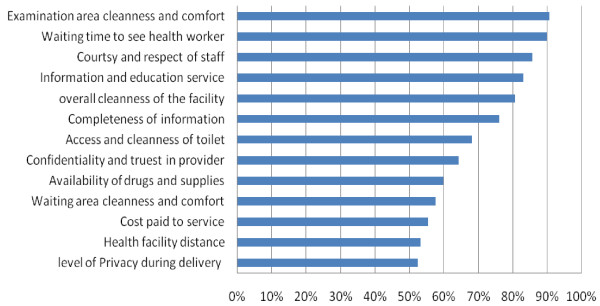
**Major dimensions of care and average satisfaction scores by postnatal mothers delivered in 3 Referral hospitals in Amhara Region, Ethiopia**.

Of all satisfaction levels, client privacy related satisfaction (46.7%), health facility distance related satisfaction (51.4%), and amount of cost paid related satisfaction (52.7%) were the first three least values (Figure 1).

Results of multivariate stepwise logistic regression indicated that health facility related factors and health providers' characteristics were important predicators of the overall maternal satisfaction.

Women's satisfaction with delivery care was associated with wanted status of the pregnancy [AOR = 2.2,95%CI:1.2,3.93)], favourable immediate maternal condition after delivery [AOR = 2.1,95%CI: 1.12,3.93)], short waiting time [AOR = 2.9, 95%CI: 1.14,7.58)], perceived availability of waiting area [AOR = 6.3,95%CI:3.33,11.88)], care providers measure taken to assure privacy during examinations [AOR = 2.1, 95%CI: 1.13,3.83)], and amount of cost paid for service [AOR = 1.9 95% CI:1.15,3.60)] (Table 3 and 4).

**Table 3:** Socio-demographic characteristics associated with mothers' satisfaction in referral hospitals of Amhara Region, Ethiopia, September-November, 2009

	Satisfied	Unsatisfied	COR (95% CI)	AOR (95% CI)
**Age (in years)**				
< 20 years	20(4.8)	17(4.1)	1.00*	1.00*
20-34 years	216(51.8)	134(32.1)	1.4(0.69,2.71)	0.7(0.28,1.99)
35-49 years	22(5.3)	8(1.9)	2.3(0.83,6.59)	1.5(0.35,6.21)
**Marital Status**				
Single	19(4.6)	20(4.8)	1.00*	1.00*
Married	236(56.6)	136(32.6)	1.8(0.94,3.54)	0.5(0.14,1.58)
Divorced	3(0.7)	3(0.7)	1.1(0.19,5.87)	1.4(0.13,15.88)
**Religion**				
Orthodox	173(41.6)	129(31.0)	1.00*	1.00*
Muslim	79(19.0)	28(6.7)	2.1(1.29,3.43)**	1.8(0.97,3.27)
protestant	5(1.2)	2(0.5)	1.9(0.36,9.76)	1.1(0.13,10.28)
**Educational Status**				
No formal education	79(18.9)	49(11.8)	1.00*	1.00*
Grade 1 - 6	42(10.1)	27(6.5)	1.0(0.53,1.76)	0.9(0.39,2.32)
Grade 7 - 12	91(21.8)	67(16.1)	0.8(0.52,1.36)	1.6(0.66,3.95)
Diploma and above	46(11.0)	16(3.8)	1.8(0.91,3.49)	1.8(0.49,6.72)
**Occupation**				
Governmental employee	57(14.5)	29(7.3)	1.00*	1.00*
Merchant	32(8.1)	19(4.8)	0.9(0.42,1.77)	0.9(0.29,2.72)
Farmer	52(13.2)	26(6.6)	1.0(0.53,1.95)	1.5(0.40,5.34)
House wife	85(21.5)	50(12.7)	0.87(0.49,1.53)	1.5(0.55,3.96)
Student	17(4.3)	25(6.3)	0.35(0.16,0.74)	0.6(0.20,2.03)
**Economic status (monthly income)**				
≤ 765ETB(47.8USD)	134(32.1)	75(18.0)	1.2(0.82,1.80)	
> 765 ETB(47.8USD)	124(29.8)	84(20.1)	1.00*	
**Residence**				
Urban	185(44.4)	111(26.6)	1.1(0.71,1.69)	
Rural	73(17.5)	48(11.5)	1.00*	
**Perceived presence of waiting area**				
Yes	221(53.0)	50(12.0)	13.0(8.03,21.11)	6.3(3.33,11.88)
No	37(8.9)	109(26.1)	1.00*	1.00*
**Mode of visit**				
Referral	104(24.9)	59(14.2)	1.1(0.76,1.72)	
Not referral	154(36.9)	100(24.0)	1.00*	
**Waiting time before seeing a doctor or a nurse**				
≤ 1 hour	247(59.2)	135(32.4)	4.0(1.89,8.40)	2.9(1.14,7.58)
> 1 hours	11(2.6)	24(5.8)	1.00*	1.00*
**Privacy during examinations**				
yes	179(42.9)	40(9.6)	6.7(4.32,10.52)	2.1(1.13,3.83)
No	79(18.9)	119(28.6)	1.00*	1.00*
**Amount paid to the service**				
≤ 157 ETB	154(36.9)	68(16.4)	2.0(1.33,2.96)	1.9(1.15,3.60)
> 157 ETB	104(24.9)	91(21.8)	1.00*	1.00*

*Reference group.

**Table 4:** Obstetric experiences associated with mothers' satisfaction in referral hospitals of Amhara Region, Ethiopia, September -November, 2009(n = 417)

Obstetric experiences	Satisfied	Unsatisfied	COR(95% CI)	AOR (95%CI)
**Wanted status of pregnancy**				
Wanted	212(50.9)	96(23.0)	3.0(1.93,4.74)*	2.2(1.20,3.93)*
			*	*
Unwanted	46(11.0)	63(15.1)	1.00*	1.00*
**Mode of delivery**				
SVD	142(34.0)	82(19.7)	1.7(1.04,2.77)*	1.2(0.50,2.69)
			*	
Assisted delivery	70(16.8)	32(7.7)	2.1(1.19,3.84)*	1.5(0.60,3.93)
			*	
Caesarean section	46(11.0)	45(10.8)	1.00*	1.00*
**Maternal condition**				
Normal	226(54.2)	102(24.4)	3.9(2.41,6.46)*	2.1(1.12,3.93)*
			*	*
With complication	32(7.7)	57(13.7)	1.00*	1.00*
**Fetal outcome**				
Lived	232(55.6)	142(34.1)	1.1(0.56,2.04)	
Died	26(6.2)	17(4.1)	1.00*	
**ANC follow up**				
Yes	227(54.4)	124(29.8)	2.1(1.22,3.51)*	1.3(0.58,3.03)
			*	
No	31(7.4)	35(8.4)	1.00*	1.00*

*Reference group.

**Figure 2:**
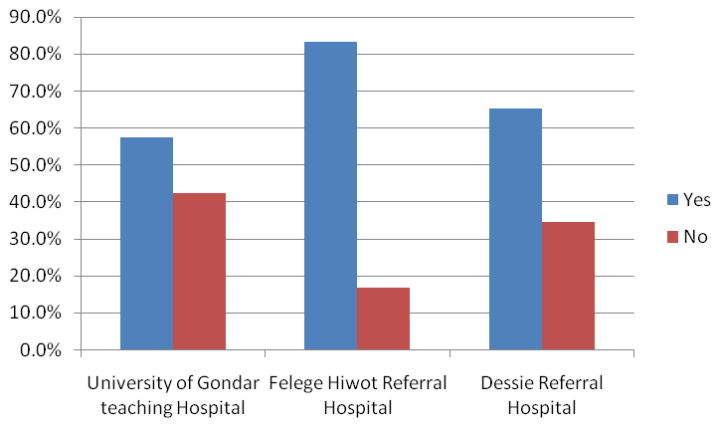
**Will you recommend the hospital to family and friends?**.

Figure 2 displays responses to questions exploring the willingness of delivering mothers to recommend the hospital to family or friend. Overall, 69.1% of women were very likely to recommend the hospital where they delivered to others. Only 57.5% of women who deliver at University of Gondar Teaching and Referral Hospital were willing to recommend the hospital to a family or a friend. On the contrary Felege Hiwot Referral Hospital was recommended by 83.3% of the mothers who delivered there. This shows a statistically significant difference with X^2^ of 24.1 and p < 0.001. Thinking about their experience, only 287(68.8%) of the delivering mothers are likely to deliver in the hospital where they deliver again.

## Discussion

This paper presents a study that estimated the level of mothers' satisfaction with referral hospitals' maternity care in Amhara Region of Ethiopia. The overall proportion of mothers who were satisfied with delivery care in this study was 61.9%. This percentage is very low compared to other studies in developing countries - 92.5% in Côte d'Ivoire [18] but it is comparable to a study in Nairobi, Kenya-56% [11] and greater than a study in Sri Lanka 48% [12]. This variation may be because of a real difference in quality of services provided, expectation of mothers or the type of health facilities. Studies conducted in Canada [19], Kenya [11] and Sir Lanka [12] suggest that services dealing with referred patients or complicated deliveries yield less satisfaction.

In this study delivering mothers satisfactions was predicted by wanted status of the pregnancy, immediate maternal condition after delivery, waiting time before seen by a health worker, perception about the waiting area for mothers and relatives, health professionals measure taken to assure privacy during maternal examinations, and service cost paid. This finding is consistent with other studies in Africa [11,12,18].

Mothers who wanted their pregnancy were more likely to be satisfied than mothers who did not. Similar finding was reported in Kenya by Bazent and her colleagues [11]. The study also showed that mothers without complication were more likely to be satisfied than mothers with complication. Women who experience no complications may be happy that they survived and this may result satisfaction with care [11].

In studies conducted in Oromia and Afar Regions of Ethiopia, mothers were complaining payment for service, inadequate privacy and unfriendly attitude of care providers [7,20]. Similarly, in this study the researchers found privacy and cost incurred for service to be associated with mothers' dissatisfaction. Mothers who reported privacy during physical examination were more satisfied than those who perceived absence of privacy. This reflects there is privacy breach which should be improved. The mothers' level of satisfaction was also related to the amount of money paid for service as mothers who paid less than or equal to157 ETB were more satisfied than those who paid greater than157 ETB. This might be due to the low socio economic status of the mothers or/and mothers may have paid unexpected costs such as cost for travel and charge for supplies.

Our finding showed that nearly two-thirds of all women were very likely to recommend the delivery care facility to friends and family. Though this suggests that the hospitals are providing an acceptable quality of care, there is room for substantial improvement at University of Gondar Teaching and Referral Hospital.

The findings of this study had limitations. First, data are restricted to delivery experience to referral hospital thereby limiting generalization to the overall health facility experience of childbirth by women. Second, potential response biases often present in patient satisfaction studies related to social desirability. We tried to minimize this bias by interviewing mothers in a separate room by trained nurses who are not affiliated with the facilities studied.

## Conclusions

The overall satisfaction of hospital delivery services in this study is found to be suboptimal. The study strongly suggests that more could be done to assure that services provided are more patient centered.

This study also revealed several constraints in the provision of delivery care services which can be implied as areas of possible improvement, including poor maintenance of clients' privacy, longer waiting time, and unavailability of waiting areas.

## Competing interests

The authors declare that they have no competing interests.

## Authors' contributions

AT wrote the proposal, participated in data collection, analyzed the data and drafted the paper. DTZ and YK approved the proposal with some revisions, participated in data analysis and revised subsequent drafts of the paper. All authors read and approved the final manuscript.

## Pre-publication history

The pre-publication history for this paper can be accessed here:

http://www.biomedcentral.com/1471-2393/11/78/prepub
